# The effect of maternal near miss on adverse infant nutritional outcomes

**DOI:** 10.6061/clinics/2016(10)07

**Published:** 2016-10

**Authors:** Dulce M Zanardi, Erly C Moura, Leonor P Santos, Maria C Leal, Jose G Cecatti

**Affiliations:** I University of Campinas, School of medicine, Department of Obstetrics and Gynecology, Campinas/SP, Brazil; II FIOCRUZ, National School of Public Health, Researcher scholarship, Rio de Janeiro/RJ, Brazil; III University of Brasília, School of Health Sciences, Department of Public Health, Brasília/DF, Brazil; IV FIOCRUZ, National School of Public Health, Rio de Janeiro/RJ, Brazil

**Keywords:** Severe Maternal Morbidity, Near Miss, Healthcare, Nutrition Disorders, Breastfeeding

## Abstract

**OBJECTIVES:**

To evaluate the association between self-reported maternal near miss and adverse nutritional status in children under one year of age.

**METHODS:**

This study is a secondary analysis of a study in which women who took their children under one year of age to the national vaccine campaign were interviewed. The self-reported condition of maternal near miss used the criteria of Intensive Care Unit admission; eclampsia; blood transfusion and hysterectomy; and their potential associations with any type of nutritional disorder in children, including deficits in weight-for-age, deficits in height-for-age, obesity and breastfeeding. The rates of near miss for the country, regions and states were initially estimated. The relative risks of infant adverse nutritional status according to near miss and maternal/childbirth characteristics were estimated with their 95% CIs using bivariate and multiple analyses.

**RESULTS:**

The overall prevalence of near miss was 2.9% and was slightly higher for the Legal Amazon than for other regions. No significant associations were found with nutritional disorders in children. Only a 12% decrease in overall maternal breastfeeding was associated with near miss. Living in the countryside and child over 6 months of age increased the risk of altered nutritional status by approximately 15%, while female child gender decreased this risk by 30%. Maternal near miss was not associated with an increased risk of any alteration in infant nutritional status.

**CONCLUSIONS:**

There was no association between maternal near miss and altered nutritional status in children up to one year of age. The risk of infant adverse nutritional status was greater in women living in the countryside, for children over 6 months of age and for male gender.

## INTRODUCTION

For many centuries, the care provided to women during pregnancy was not perceived as a health issue but was considered part of the daily domestic life experienced by mothers and midwives. Only in the twentieth century has this subject become a global priority in the context of public health 1.

More recently, efforts have been employed to reduce maternal and infant mortality worldwide. The overall number of maternal deaths has decreased widely from 376,034 in 1990 to 292,982 in 2013. The annual variation in maternal mortality ratio per 100,000 live births (MMR) was -0.3% (-1.1 to 0.6) from 1990 to 2003 and -2.7% (-3.9 to -1.5) from 2003 to 2013. The MMR decreased considerably in South Asia, East Asia and Southeast Asia between 1990 and 2013 but increased in sub-Saharan Africa during the 1990s. A global analysis suggested that only 16 countries had probably achieved the Millennium Development Goal (MDG5), which was to establish a 75% reduction in maternal mortality by 2015 2,3. Public policies are fundamental because maternal mortality is strongly associated with poverty, undernutrition and low social development 2.

Furthermore, infant mortality has also shown important variations. In 2013, 6.3 million children under 5 years of age died, with a reduction of 64% compared with 17.6 million in 1970. In 2013, infant mortality rates ranged from 152.5/1,000 live births in Guinea-Bissau to 2.3 per 1,000 live births in Singapore. The Millennium Development Goal 4 (MDG4) established a reduction in infant mortality by two-thirds from 1990 to 2015. Only an estimated 27 developing countries have achieved this goal. In these studies, the factors reported as most strongly related to the reduction in infant mortality were improving family income, better education, low number of births, control of HIV infection and some other factors 3,4.

Brazil remains a country where social inequalities cause deaths in both pregnant women and children that are far from the desired levels. In addition to death, other morbid conditions in both the mother and child should be avoided because they may result in short-term, medium-term and long-term health consequences for these individuals.

In terms of maternal health, the World Health Organization (WHO) has recognized and defined the importance of the “Maternal Near Miss” (MNM) since 2009. The WHO proposed that a group of criteria should be employed to identify MNM 5. The MNM criteria can only be used prospectively and involve a combination of clinical, laboratory and management information. As a result, these criteria were later simplified for routine database use and self-evaluation, including ICU admission, blood transfusion, hysterectomy and eclampsia 6.

Concerning child health, many actions have been taken to decrease preventable death, including the management of infant malnutrition, which is associated with mortality. There is a global trend toward infant undernutrition, while obesity rates increase. Infant obesity, which is an increasingly common condition, should also be avoided. However, undernutrition still threatens infant health in low- and middle-income countries 7.

Maternal and infant mortality rates remain elevated in Brazil, with important regional differences. Therefore, efficient public policies are pivotal. Combined actions were adopted that enabled the country to achieve the MDG of decreasing infant mortality rates in 2013 8. However, the same has not occurred for maternal mortality. A possible correlation between maternal morbidity/mortality and potential adverse effects on child health has not been fully explored yet. Such an association may be the basis for guidance on priorities for execution of actions and allotment of financial resources that support these actions.

In 2010, the Department of Science and Technology of the Brazilian Ministry of Health (Decit/MS) planned a study to search for elements to reduce regional inequalities in mother and child health. This study, termed the Neonatal Call study, also aimed to assess the quality of care provided to mother and child. The data from this study are serving as a new tool to reduce infant mortality and complications occurring associated with pregnancy in the Brazilian Northeastern Region and Legal Amazon 9. Thus, the aim of the current study was to evaluate the association between adverse nutritional conditions in the child and the occurrence of MNM identified among the women from the Legal Amazon and Northeast of Brazil who participated in the above-mentioned study.

## MATERIALS AND METHODS

Neonatal Call was a cross-sectional study. Data collection occurred in the first phase of the infant vaccine campaign on June 12, 2010. Data were obtained from the mothers of children under one year of age. These mothers were present at the basic health services and were selected to participate in the research. In the study, 252 municipalities (98%) participated of the 256 that signed the pact to reduce infant mortality, involving 17 federation units in the Legal Amazon and Northeast of Brazil 9.

Sample size calculation considered a predicted prevalence of 22% for “any self-reported complication during childbirth” in accordance to data derived from the Brazilian National Demographic Health Survey 2006-2007 10. As a result, 412 pairs of mother and child were included with a simple random design in each sample domain (9). All capitals and municipalities containing the highest number of births and infant deaths in the region were included to have a representative sample of each region. In total, the sample consisted of 23,399 mother-child pairs. The search for vaccinations was poorer than expected and 16,863 pairs of mother and child under one year of age were interviewed, achieving more than 70% of the goal in most domains 9.

The current study is a secondary database analysis of the larger study. It focused on adverse nutritional status in children under one year of age and its potential association with the occurrence of self-reported MNM at the time of birth (exposure), in a retrospective cohort approach. For analysis, the inclusion criteria were children under one year of age, those living within the municipality of the immunization clinic, and those who were not twins or had not been adopted. If the mother had two children under one year of age, the youngest child was selected. A data collection instrument was developed to fulfill the aims of the study. It was previously tested in two regions by regional coordinators. Questions included information on the sociodemographic characteristics of the woman and child, prenatal care, delivery and the postpartum period, complications associated with birth, perinatal outcomes, vaccinations, growth surveillance and feeding habits 9.

Pregnancy complications were self-reported by the women and defined as any hospitalization due to medical conditions during pregnancy or delivery. Participants were specifically questioned about the occurrence of severe maternal complications and procedures performed, including ICU admission, mechanical ventilation, eclampsia, blood transfusion, infection, hysterectomy and prolonged hospitalization. The procedure using this specific data collection instrument had already been validated in the country 6. MNM cases were identified according to the pragmatic criteria previously defined by the WHO in 2009, e.g., ICU admission, eclampsia, hysterectomy or blood transfusion 11.

Infant anthropometric measurements (weight and height) were collected in duplicate for each child on the day of vaccination. Measurements were made by two anthropometry specialists trained for the study. The results were written in different forms, according to the criteria for procedures defined by the WHO in 2006 12. Deficits in weight and height for age were considered for z scores of respective indicators below 2 standard deviations. Obesity was defined as a z score of weight-for-height and BMI-for-age above 2 standard deviations 12. Data were recorded on the child's card and the family was informed about the nutritional status.

The study protocol was approved by the Institutional Review Board of the National School of Public Health/Oswaldo Cruz Foundation (Fiocruz, Rio de Janeiro, Brazil) through a letter of approval (CEP/ENSP 56/10).

### Data analysis

Initially, the occurrence of MNM (% and 95% CI) was estimated for the total study sample and the respective states that compose the Legal Amazon and Northeast region of Brazil, using pragmatic MNM criteria. Different parameters of adverse nutritional status in children under one year of age (deficit in weight-for-age, deficit in height-for-age, obesity, any breastfeeding and exclusive breastfeeding until 6 months) were compared between mothers who had MNM and those without any reported severe morbidity using risk ratio and 95% CI. Later, the risk ratio (and 95% CI) was also calculated for any adverse infant nutritional status, regarding maternal sociodemographic characteristics, prenatal care, childbirth care, perinatal care and the occurrence of MNM. Finally, the adjusted risk ratio (and 95% CI) of the variables that were independently associated with any adverse nutritional status were calculated by multiple analyses.

## RESULTS

The Neonatal Call study interviewed 16,863 women from 17 states of the Northeast region and Legal Amazon. Of these women, 12,897 women met the inclusion criteria and comprised the sample that is currently analyzed. These women had complete information on the occurrence of pregnancy-related complications and infant nutritional parameters. Table 1 shows that the proportion of self-reported MNMs for the entire sample was 2.9%, without a difference between the Legal Amazon (3.4%) and Northeast (2.7%). Among the pragmatic criteria of MNM, a very similar variation in proportion was observed between the different regions for ICU admission, eclampsia, blood transfusion and hysterectomy. Hysterectomy was reported less frequently and by only 0.1% of the women in the Northeast. The most commonly reported pragmatic criterion was eclampsia for both regions, with similar proportions (1.8% in the Legal Amazon, 1.5% in the Northeast).

Table 2 shows the estimated risk of different alterations in nutritional status (assessed as a weight-for-age deficit, a length-for-age deficit and obesity), any maternal breastfeeding and exclusive maternal breastfeeding until 6 months in different regions, which is reported along with the occurrence of MNM. Women with MNM breastfed 12% less than those without severe complications. In the Northeast, this proportion was 17% *versus* 6% in the Legal Amazon. No significant differences were found for exclusive maternal breastfeeding until 6 months of age. The most common nutritional alteration was a deficit in height, followed by a deficit in weight, in both regions. However, there were no significant differences between women reporting MNM and the remaining women.

Table 3 shows the estimated risks of any alteration in infant nutritional status according to some maternal and obstetric characteristics. Living in the countryside and the child being over 6 months old increased the risk by approximately 15%. In contrast, in females, the risk of nutritional alteration was 30% less than in males. The remaining maternal or obstetric characteristics that were evaluated (maternal age, maternal schooling, ethnicity, being head of the family, adequacy of prenatal care and the occurrence of MNM) did not significantly increase the risk of nutritional status alteration in children. In the multivariate analysis shown in Table 4, the same factors (living in the countryside, child older than 6 months and child gender) were independently associated with risk of altered infant nutritional status, confirming the results of bivariate analysis.

## DISCUSSION

The aim of the current study was to correlate the occurrence of MNM with nutritional status in children up to one year of age, including weight-for-height deficit, length-for-height deficit, and obesity. In addition, any breastfeeding and exclusive maternal breastfeeding practices until 6 months were measured in these children. The only factor associated with the self-report of MNM was a decrease in any maternal breastfeeding, which was mainly observed in the Northeast region. In contrast, the occurrence of adverse nutritional status in children was associated with the place of residence (living in the countryside), the child being over six months of age or being male. These results did not confirm the initial hypothesis that the occurrence of MNM could have a negative effect on child health in the first year of life, at least not on infant nutritional status.

Intuitively, women suffering from MNM conditions may also have had multiple difficulties, leading to suboptimal maternal breastfeeding practices, as we observed in this study. In 2003, the WHO recommended maternal exclusive breastfeeding until 6 months of age 13. Early weaning causes short-term, medium-term and long-term consequences, both in low-income and high-income families. The lack of breastfeeding contributes to an increase in infant mortality, hospitalization, increases in gastrointestinal and respiratory infection, obesity and diabetes as a child and cardiovascular disease as an adult. In women who do not breastfeed, there is an increase in the incidence of breast cancer. Our study did not have a sufficient follow-up period to confirm any of these conditions. The cost of weaning should include acute and chronic diseases in mothers and children 14. In a recent study of Brazilian children, maternal breastfeeding was associated with improved cognitive performance 15. The current study was not aimed at correlating the weaning process with episodes of infection in children or other possible consequences, such as neurological and cognitive development. However, factors that may reduce the rates of maternal breastfeeding should be avoided because maintaining an adequate breastfeeding practice is fundamental for infant health.

The concept of MNM is relatively new. The term has been used with some frequency to broaden the discussion of the possible effects of severe maternal morbidity on women's health in different situations. Theoretically, the great advantage of using the concept of MNM is that a woman who survived a complicated delivery and postpartum period can report the conditions in which the MNM occurred and its short-term, medium-term and long-term effects. Thus, constraints on women's healthcare can be identified and specific strategies for the reduction in MNM can be established 16. Although the maternal mortality rate has significantly decreased in the last two decades, Brazil still needs to reduce the high maternal mortality ratio to achieve MDG5 17. The Ministry of Health reported a maternal mortality ratio ranging from 58.7-64.8 per 100,000 live births 18. In a study published in 2015 using the Neonatal Call database, MNMs had a prevalence of 37.5 per 1,000 live births in the Legal Amazon and Northeast of Brazil, predominating in the Legal Amazon 9,19. However, the effects of severe maternal morbidity and its consequences for the newborn infant and child are still not fully elucidated and the current study failed to confirm the hypothesis of adverse nutritional indicators, at least in the child's first year.

The current study also showed that girls had a 30% lower risk of having some alteration in nutritional status. The risk of some type of infant nutritional deficit increased by approximately 15% in children living in the countryside and in those over six months of age. Children living in the countryside may have had a higher risk of altered nutritional status because their mothers had more severe obstetric complications or they had difficult access to facilities that could resolve these complications. We have no available data from other national studies that lend support to these findings. Studies that were conducted in two states in Nigeria and investigated the association between pregnancy conditions and nutrition showed that female children had a higher chance of presenting with malnourishment in the first year of life, which suggests different levels of valuing children in the society, depending on gender and the local culture 20.

A study on nutritional deficiency was conducted in Pelotas, in the south of Brazil. Three cohorts of children under one year of age (born in 1984, 1993 and 2004) were analyzed. The study showed that, after the prevalence of nutritional deficiency declined between 1982 and 1993, it stabilized between 1993 and 2004. During the entire study period, all deficiencies decreased in prevalence. In contrast, the prevalence of obesity increased. Low height for age was strongly associated with family income. The group with low family income, below one minimal wage, was the only group to show a significant reduction in low height-for-age during the study period. There was a major improvement in the reduction of nutritional deficiency in the first half of the study period, although social inequalities persisted. It is imperative to fight against undernutrition, particularly among the most deprived children in the population. At the same time, overweight should also be managed. It has become increasingly prevalent across all social classes 21.

Historically, childhood undernutrition has been associated with low family income and living in rural areas. However, in the recent years, obesity has also been reported in rural areas and low-income families. Childhood is a very important period for physical and cognitive development. Malnourishment decreases the growth potential of a society. Undernourished children will usually become shorter adults, with less working capacity. Undernutrition also contributes to a reduction in cognitive development, failure at school, low performance and low level of school education. Childhood obesity also reduces a potential contribution to society and results in adults who suffer from chronic diseases 22,23.

Brazil has still not overcome the problems related to early weaning and nutritional deficiencies. Fighting against adverse factors should remain prioritized to favor adequate child growth. Exclusive breastfeeding until 6 months of age and breastfeeding with complementary food until two years of age, as recommended by the WHO in 2003 13, is still inadequate worldwide. In more deprived communities where infants are undernourished, it is a health issue to be combatted 14,20,24–26. In the Neonatal Call study, breastfeeding in the first hour, which can improve maternal breastfeeding index, occurred in 64% of the cases 9. Exclusive breastfeeding until 6 months occurred in 22.3% of children and the median duration of exclusive maternal breastfeeding was 64 days. Breastfeeding was accessible to 80.6% of infants under one year of age, and all indicators were more favorable in the Legal Amazon than in the Northeast of the country 9. Exclusive breastfeeding may increase even further in Northeastern Brazil and is a strategical target that should be prioritized. In women with MNMs, there was inadequate breastfeeding practice, considering that the study was extended to the first year of age. However, postpartum maternal health status may have led to separation between mother and child and could have reduced the chance of exclusive breastfeeding until one year of age 27.

The current study also showed that some nutritional deficiencies in children were associated with place of residence, particularly in the countryside. In the poorest African regions, deficiencies are most commonly encountered in children from rural populations with less access to healthcare facilities 23–26. Another study focused on inhabitants of rural areas of the USA in search of future health priorities for the 2020s. The research showed that the priorities were identical to those observed in the previous decade and highlighted that access to health continues to be a major priority 28. Explanations for the higher risk of any nutritional alteration in children living in rural zones could include the severity of their mothers' complications or the difficulty associated with accessing healthcare to resolve these complications. Limited access to healthcare remains an important factor for the poor performance in health globally.

The current study failed to show any association between the occurrence of MNM during pregnancy and adverse nutritional status in children under one year of life, as previously hypothesized. This association may not have been demonstrated due to some limitations of the current study. First, this study was not designed with this specific purpose because it was a secondarily planned analysis. Furthermore, the study design may not have been favorable for recognizing the more severe MNM cases because the study covered mothers and children on the day of the vaccine campaign. More severe cases may not have been identified in the study. Mothers and children suffering from the worst MNM sequelae, could have theoretically been unable to participate in the campaign, due to adverse health conditions. Other designs should be proposed to improve the identification of more severe cases. These studies may establish whether there is a correlation between MNM and adverse nutritional status in children through a prospective cohort study. An inadequate breastfeeding practice was the only identified factor associated with MNM in these women. Thus, other methodological approaches are recommended to further explore this potential association among MNM, altered nutritional status and child health during the first year of life.

### Significance

What is already known on this subject?

Although women who have had any severe complication associated with pregnancy and childbirth would intuitively be more likely to have children with a higher likelihood of death, morbidity and long-term adverse effects on health and nutrition status, this association has never been assessed before, at least when considering the occurrence of a standardly defined maternal near miss event.

What this study adds?

The current study did not identify any adverse nutritional status in children associated with the occurrence of maternal near miss events; however, it showed a lower prevalence of women who had breastfed in these conditions. A prospective design would better address this possible relationship.

## AUTHOR CONTRIBUTIONS

Zanardi DM and Cecatti JG conceived of the current analysis. The data had already been collected for the main study. Zanardi DM and Cecatti JG drafted the analysis plan, which was performed by Moura EC. Zanardi DM drafted the manuscript. All authors discussed the analysis results and then reviewed, suggested and agreeded on the content of the final version of the manuscript.

## Figures and Tables

**Table 1:** Occurrence of maternal near miss and its main determinants (pragmatic criteria) in the Legal Amazon and Northeast regions of Brazil. Neonatal Call, Brazil, 2010.

Places	Sample	ICU admission			Eclampsia			Blood transfusion			Hysterectomy			MNM		
	n	n	%	CI _95%_	n	%	CI _95%_	n	%	CI _95%_	n	%	CI _95%_	n	%	CI _95%_
**Legal Amazon**	**5394**	**34**	**0.6**	**(0.3-0.8)**	**91**	**1.8**	**(1.3-2.2)**	**52**	**0.9**	**(0.6-0.0)**	**17**	0**.3**	**(0.1-0.5)**	**181**	**3.4**	**(2.8-4.0)**
Acre	469	3	0.5	(0.0-1.1)	14	3.1	(1.4-4.9)	6	1.1	(0.2-0.0)	4	0.7	(0.0-1.4)	25	5.1	(3.0-7.2)
Amapá	513	2	0.4	(0.0-1.0)	14	2.5	(1.2-3.8)	3	0.7	(0.0-0.0)	0			17	3.1	(1.6-4.6)
Amazonas	1090	9	0.7	(0.2-1.2)	22	2.0	(1.1-2.8)	12	1.2	(0.5-0.0)	4	0.3	(0.0-0.6)	45	3.9	(2.7-5.1)
Mato Grosso	963	2	0.1	(0.0-0.3)	11	1.3	(0.5-2.1)	8	0.9	(0.2-0.0)	0			21	2.3	(1.3-3.4)
Pará	626	5	0.8	(0.1-1.5)	9	1.9	(0.6-3.2)	6	0.8	(0.1-0.0)	4	0.6	(0.0-1.3)	22	3.7	(2.1-5.3)
Rondônia	414	1	0.2	(0.0-0.5)	3	0.5	(0.0-1.1)	0			0			4	0.7	(0.0-1.4)
Roraima	390	5	1.3	(0.2-2.4)	10	2.6	(1.0-4.1)	8	2.1	(0.6-0.0)	3	0.8	(0.0-1.6)	22	5.6	(3.4-7.9)
Tocantins	929	7	0.7	(0.2-1.2)	8	0.8	(0.2-1.3)	9	1.1	(0.3-0.0)	2	0.2	(0.0-0.4)	25	2.6	(1.5-3.7)
**Northeast**	**7503**	**52**	**0.6**	**(0.3-0.8)**	**104**	**1.5**	**(1.1-1.9)**	**70**	**0.8**	**(0.6-0.0)**	**15**	**0.1**	**(0.1-0.2)**	**212**	**2.7**	**(2.2-3.2)**
Alagoas	22	0			1	4.5	(0.0-13.3)	0			0			1	4.5	(0.0-13.3)
Bahia	670	3	0.4	(0.0-0.8)	13	1.8	(0.7-2.9)	5	0.7	(0.1-0.0)	2	0.2	(0.0-0.5)	19	2.6	(1.3-3.9)
Ceará	1237	5	0.4	(0.0-0.8)	21	1.7	(0.9-2.6)	14	1.1	(0.5-0.0)	1	0.0	(0.0-0.1)	36	2.9	(1.9-4.0)
Maranhão	852	3	0.2	(0.0-0.5)	10	1.1	(0.3-1.9)	5	0.3	(0.0-0.0)	3	0.2	(0.0-0.4)	18	1.5	(0.7-2.4)
Paraíba	1563	14	0.7	(0.3-1.1)	11	0.7	(0.2-1.2)	16	0.8	(0.4-0.0)	2	0.1	(0.0-0.3)	42	2.2	(1.5-3.0)
Pernambuco	854	8	1.1	(0.0-2.3)	10	1.7	(0.3-3.0)	11	1.4	(0.4-0.0)	0			27	4.2	(2.1-6.2)
Piauí	861	11	1.3	(0.5-2.0)	9	1.0	(0.3-1.8)	7	0.8	(0.2-0.0)	1	0.1	(0.0-0.4)	24	2.8	(1.6-3.9)
RG do Norte	740	5	0.6	(0.1-1.1)	12	1.6	(0.6-2.5)	5	0.5	(0.0-0.0)	4	0.4	(0.0-0.8)	20	2.5	(1.3-3.6)
Sergipe	704	3	0.3	(0.0-0.6)	17	2.0	(1.0-3.1)	7	0.8	(0.2-0.0)	2	0.2	(0.0-0.6)	25	3.0	(1.7-4.2)
**Total**	**12897**	**86**	**0.6**	**(0.4-0.8)**	**195**	**1.6**	**(1.3-1.9)**	**122**	**0.9**	**(0.7-0.0)**	**32**	**0.2**	**(0.1-0.3)**	**393**	**2.9**	**(2.5-3.3)**

**Table 2:** Estimated risks of altered nutritional status of children according to the occurrence of maternal near miss during pregnancy by region. Neonatal Call. Brazil, 2010.

Children nutritional status		Sample	%	MNM	No severe morbidity	RR (95% CI)
**Deficit of weight/age**	Total	12873	4.4	4.3	95.7	1.49 (0.92-2.40)
	Legal Amazon	5382	5.0	4.2	95.8	1.25 (0.69-2.26)
	Northeast	7491	4.2	4.4	95.6	1.63 (0.83-3.21)
**Deficit of length/age**	Total	12819	9.7	3.7	96.3	1.29 (0.86-1.96)
	Legal Amazon	5363	9.6	3.8	96.2	1.14 (0.66-1.97)
	Northeast	7456	9.7	3.7	96.3	1.39 (0.80-2.42)
**Obesity**	Total	12787	9.2	2.7	97.3	0.90 (0.60-1.35)
	Legal Amazon	5342	9.4	4.1	95.9	1.23 (0.72-2.11)
	Northeast	7445	9.2	1.9	98.1	0.70 (0.39-1.25)
**Any breastfeeding**	Total	12873	80.8	2.6	97.4	**0.88 (0.80-0.96)**
	Legal Amazon	5374	85.2	3.1	96.9	0.94 (0.86-1.03)
	Northeast	7499	78.7	2.3	97.7	**0.83 (0.72-0.96)**
Exclusive breastfeeding for 6 months*	Total	6012	1.2	2.7	97.3	0.85 (0.16-4.58)
	Legal Amazon	2445	1.5	1.2	98.8	0.32 (0.04-2.36)
	Northeast	3567	1.1	2.7	96.3	1.28 (0.17-9.65)

**Table 3:** Estimated risks of some altered nutritional status of children (composite variable, including weight/length) according to some maternal and obstetric characteristics.

Factor		Sample	Children with any altered nutritional status*	Children with normal nutritional status	RR (95% CI)
**Maternal age (years)**	<20	2.538	18.9	81.1	1.00
	20 to 29	7.009	18.2	81.8	0.96 (0.84-1.10)
	≥ 30	3.191	18.5	81.5	0.98 (0.84-1.14)
**Maternal schooling (years)**	< 8	3.476	19.8	80.2	1.00
	8 to 11	3.698	17.8	82.2	0.90 (0.79-1.03)
	≥ 11	5.609	18.2	81.8	0.92 (0.81-1.04)
**Maternal ethnicity**	white	2.594	18.4	81.6	1.00
	black	9.765	18.5	81.5	1.01 (0.89-1.15)
	indigenous and Asian	479	18.7	81.3	1.02 (0.77-1.35)
**Head of family**	mother	3.261	18.5	81.5	1.00
	other	9.532	18.5	81.5	1.00 (0.88-1.12)
**Living place**	capital	6.44	17.0	83.0	1.00
	Countryside	6.457	19.5	80.5	**1.15 (1.05-1.26)**
**Adequacy of PNC**	adequate	405	14.0	86.0	1.00
	partially adequate	5.036	18.5	81.5	1.32 (0.94-1.86)
	inadequate	7.29	18.5	81.5	1.32 (0.94-1.85)
**Adequacy of delivery care**	adequate	72	17.8	82.2	1.00
	partially adequate	9.783	18.0	82.0	1.01 (0.53-1.94)
	inadequate	1.754	19.5	80.5	1.09 (0.56-2.12)
**Child gender**	male	6.579	21.6	78.4	1.00
	female	6.318	15.2	84.8	**0.70 (0.63-0.78)**
**Child age**	<6 months	6.876	17.2	82.8	1.00
	≥6 months	6.021	20.0	80.0	**1.16 (1.05-1.28)**
**Maternal Near Miss**	no	12.504	18.4	81.6	1.00
	yes	393	20.9	79.1	1.14 (0.86-1.50)

* includes obesity and deficits of weight and length

**Table 4:** Factors that are independently associated with any altered infant nutritional status* by multivariate analysis.

Factor		RR_adj_ (95% CI)	*p*-value
**Maternal age (years)**	<20	1.00	0.997
	20 to 29	0.99 (0.86-1.15)	
	≥30	1.00 (0.85-1.17)	
**Maternal schooling (years)**	<8	1.00	0.473
	8 to 11	0.92 (0.80-1.06)	
	≥11	0.95 (0.83-1.09)	
**Maternal ethnicity**	white	1.00	0.606
	black	1.03 (0.90-1.18)	
	indigenous and Asian	1.08 (0.80-1.45)	
**Head of family**	mother	1.00	**0.834**
	other	**0.99 (0.87-1.12)**	
**Place of residence**	capital	1.00	0.011
	countryside	1.14 (1.03-1.27)	
**Adequacy of PNC**	adequate	1.00	0.820
	partially adequate	1.39 (0.97-2.01)	
	inadequate	1.34 (0.93-1.93)	
**Adequacy of delivery care**	adequate	1.00	0.239
	partially adequate	1.47 (0.72-2.96)	
	inadequate	1.57 (0.77-3.21)	
**Child gender**	male	1.00	**<0.001**
	female	**0.68 (0.60-0.76)**	
**Child age**	<6 months	1.00	**0.017**
	≥6 months	**1.14 (1.02-1.28)**	
**Maternal near miss**	no	1.00	0.588
	yes	1.09 (0.80-1.49)	

* includes obesity and deficits of weight and length
